# Telemedicine solutions for clinical care delivery during COVID-19 pandemic: A scoping review

**DOI:** 10.3389/fpubh.2022.937207

**Published:** 2022-07-22

**Authors:** Raheleh Ganjali, Mahdie Jajroudi, Azam Kheirdoust, Ali Darroudi, Ashraf Alnattah

**Affiliations:** ^1^Clinical Research Development Unit, Emam Reza Hospital, Mashhad University of Medical Sciences, Mashhad, Iran; ^2^Department of Medical Informatics, Faculty of Medicine, Mashhad University of Medical Sciences, Mashhad, Iran; ^3^Pharmaceutical Research Center, Mashhad University of Medical Sciences, Mashhad, Iran; ^4^Department of Health Information Technology, Faculty of Paramedicine, Mashhad University of Medical Science, Mashhad, Iran

**Keywords:** telemedicine, information technology, setting, outcomes, function, context, COVID-19

## Abstract

**Background:**

The unexpected emergence of coronavirus disease 2019 (COVID-19) has changed mindsets about the healthcare system and medical practice in many fields, forcing physicians to reconsider their approaches to healthcare provision. It is necessary to add new, unique, and efficient solutions to traditional methods to overcome this critical challenge. In this regard, telemedicine offers a solution to this problem. Remote medical activities could diminish unnecessary visits and provide prompt medical services in a timely manner.

**Objective:**

This scoping review aimed to provide a map of the existing evidence on the use of telemedicine during the COVID-19 pandemic by focusing on delineation functions and technologies, analyzing settings, and identifying related outcomes.

**Methods:**

This review was conducted following the Arksey and O'Malley framework and the Preferred Reporting Items for Systematic Reviews and Meta-Analyses Extension for Scoping Reviews (PRISMA-ScR) checklist. PubMed and Scopus databases were systematically searched based on specific eligibility criteria. The English publications included in this study focused on telemedicine systems implemented during the COVID-19 pandemic to provide clinical care services. Two independent reviewers screened the articles based on predefined inclusion and exclusion criteria. The relevant features of telemedicine systems were summarized and presented into the following four domains and their subcategories, including functionality, technology, context, and outcomes.

**Results:**

Out of a total of 1,602 retrieved papers, 66 studies met the inclusion criteria. The most common function implemented was counseling, and telemedicine was used for diagnosis in seven studies. In addition, in 12 studies, tele-monitoring of patients was performed by phone, designed platforms, social media, Bluetooth, and video calls. Telemedicine systems were predominantly implemented synchronously (50 studies). Moreover, 10 studies used both synchronous and asynchronous technologies. Although most studies were performed in outpatient clinics or centers, three studies implemented a system for hospitalized patients, and four studies applied telemedicine for emergency care. Telemedicine was effective in improving 87.5% of health resource utilization outcomes, 85% of patient outcomes, and 100% of provider outcomes.

**Conclusion:**

The benefits of using telemedicine in medical care delivery systems in pandemic conditions have been well–documented, especially for outpatient care. It could potentially improve patient, provider, and healthcare outcomes. This review suggests that telemedicine could support outpatient and emergency care in pandemic situations. However, further studies using interventional methods are required to increase the generalizability of the findings.

## Introduction

The coronavirus disease 2019 (COVID-19) pandemic has greatly challenged and overwhelmed economic and health systems, leading to the deaths of more than 1 million people, although many countries have controlled the initial outbreak, there is still the risk of resurgence (1–3). In order for continuous surveillance, risk management, disease mitigation, and complete containment, health systems need to reorganize resources and rearrange clinical services at the population level so that they could meet the public health requirements and minimize the risk of transmission by providing timely healthcare services (3, 4). To overcome this critical challenge, new, unique, and efficient solutions must be added to traditional methods. In this regard, technology offers a solution to this problem. While researchers are trying to early diagnose and treat the disease and develop vaccines for the virus, technologists have applied technology to reduce the spread of the disease and provide healthcare. Remote medical and health activities could decrease unnecessary visits and provide prompt medical services in a timely manner (1). With the development of technology and the Internet and incrementing the video-based communication potential over the last decade, a new and effective paradigm has been formed to provide telehealth and telemedicine (2, 3). Telehealth refers to the utilization of information and communication technologies (ICTs) to deliver remote healthcare-related services, while telemedicine is defined as the use of electronic data and telecommunication technologies to improve clinical healthcare delivery to patients at long distances (3–6). Given the purpose of telemedicine and the model of clinical care delivery, we preferred to use the term “telemedicine” in this article to refer to all forms of ICT-based medical care. In telemedicine, professionals may use videoconferencing to provide real-time counseling (synchronous modality) or “store-and-forward” technologies to transfer medical data (e.g., images, notes, and diagnostic test results) to healthcare providers so that they could later use them for disease diagnosis and management (asynchronous modality) (7). Telemedicine could be used as a tool to increase patients' access to quality care services in both developing and developed countries. It is specifically effective in situations where there is a barrier to receiving treatment (8). During the COVID-19 pandemic, telemedicine was the best and safest method for patients and providers to maintain their physical distance when patients needed prompt and affordable care. Various configurations, including text messages, email, smartphone applications, and wearable devices, could be applied to perform virtual visits and share information between different subjects (9). Telemedicine could become a useful asset in routine care settings and offer many benefits to the entire healthcare delivery spectrum, such as reducing resource use, enhancing access to healthcare, and reducing the risk of direct person-to-person transmission of COVID-19 (7, 8).

Many studies have been performed to evaluate telemedicine systems in pandemic situations (10–12). Some of these studies have reviewed barriers and facilitators of telehealth, and some of them have investigated telehealth services for a special field during the COVID-19 pandemic (13, 14). None of these studies have focused on clinical care, while the use of telemedicine in pandemic conditions is more critical and complex for infected patients requiring rapid interventions. This study could help healthcare managers and providers in planning and designing telemedicine in clinical care settings. This study could also help healthcare managers in evaluating telemedicine systems.

Therefore, the main purpose of this scoping review was to identify the applications of telemedicine in medical care delivery during the COVID-19 pandemic. The first objective was to characterize the functionality of telemedicine services in clinical care delivery. The second objective was to characterize the technologies used in current clinical practices. The third objective was to describe the results of telemedicine studies and their effects on clinical care.

## Methods

Review studies allow further analysis of possible gaps for potential innovation. Accordingly, we believed that a scoping review using the most recent guidelines (the Arksey and O'Malley framework and the Preferred Reporting Items for Systematic Reviews and Meta-Analyses Extension for Scoping Reviews [PRISMA-ScR] checklist) was the most appropriate method to (1) address the research questions, (2) identify related studies, (3) choose relevant studies, (4) chart data, and (5) collate and summarize results (15, 16).

### Research questions

The research questions were as follows:

- Which functionalities of telemedicine systems have been described in the context of COVID-19?
- Which technologies have been used in clinical practices?
- Which outcomes have been evaluated in clinical care during COVID-19?

### Identification of relevant studies

PubMed and Scopus databases were searched to identify potentially relevant studies published, followed by the World Health Organization (WHO) initial announcement regarding a cluster of pneumonia cases in Wuhan from 31 December 2019 to 19 September 2020. The search was conducted in the third week of September, and the collected data were exported to Microsoft Excel for screening and charting. Search terms selected for the literature search included telemedicine domains and the target pandemic context of its implementation along with Boolean operators (OR/AND). The final detailed search strategy is included in Supplementary Materials 1. Table 1 shows keywords and MeSH terms related to telemedicine and COVID-19. We defined telemedicine as the application of remote telecommunication technology to treat, diagnose, counsel, and follow-up or mentor patients in the COVID-19 context.

**Table 1:** Keywords and MeSH terms used in literature search.

**COVID-19**	**Keywords**	**Severe acute respiratory syndrome coronavirus, Wuhan coronavirus, Wuhan seafood**
		**market pneumonia virus, COVID19 virus, COVID-19 virus, coronavirus disease**
		**2019 virus, SARS-CoV-2, SARS2, 2019-nCoV**
	MeSH terms	-
Telemedicine	Keywords	Telemetry, telemedicine, mobile health, m-health, telehealth, telecare, e-health
	MeSH terms	Telemedicine

### Selection of relevant studies

Search results were screened in a reference manager by two authors (RG and MJ), and publications unrelated to the domain of this research were removed based on a review of their titles and abstracts. If the articles were not satisfactorily removed based on the information available in their title, abstract, or both, their full text was retrieved and reviewed for more clarity. Disagreements were resolved by including the articles in an in-depth analysis.

Inclusion criteria used during the article screening process were as follows: (1) studies aimed at improving at least one treatment or management outcome during the COVID-19 pandemic; (2) articles about applying telemedicine; (3) randomized studies, including quasi-experimental studies and randomized controlled trials, and non-randomized studies, including cohort, case–control, and cross-sectional studies; (4) studies published in English; (5) studies published in scientific journals; and (6) studies published from 2019 to 2020.

Exclusion criteria were as follows: (1) articles whose title, abstract, or full text were not related to COVID-19; (2) thesis, book chapters, letters to editors, editorials, short briefs, reviews or meta-analyses, case studies, conference papers, and study protocols; (3) articles whose full text was not available; (4) studies that used telemedicine in primary care; (5) survey studies that investigated attitudes toward telemedicine without implementation; and (6) studies that described only the implementation phase.

### Data charting

To chart the data, information was collected and categorized into extraction sheets according to four domains, namely, functionalities, technology, setting, and outcomes (17).

The functionality domain incorporated all aspects of the medical care process, including diagnosis, treatment, follow-up, and rehabilitation. This dimension was divided into four categories, namely, (1) counseling, (2) diagnosis, (3) monitoring, and (4) mentoring. The components of the technology dimension were grouped into two sets of variables, including synchronicity and network design. Open Internet and social networks were subcategories of network design/configuration, in which information is posted and shared.

The third domain was a setting that contained a site for providing care or needed care and was divided into three groups, namely, emergency care, outpatient care, and inpatient care.

Telemedicine outcomes were the fourth domain. They were divided into three groups, namely, healthcare resource utilization, patient, and healthcare provider outcomes.

### Data collation and summarization

Data extracted from the studies were about study sample, study type, objective, function, technology, network, sample size, outcomes, findings, and conclusion. We summarized and presented the features related to telemedicine systems.

The effect of telemedicine was defined as (1) positive (i.e., its effect was statistically significant or more than 50%) and (2) no effect (i.e., its effect was not statistically significant or < 50%).

## Results

### Selection of relevant studies

**Figure 1:**
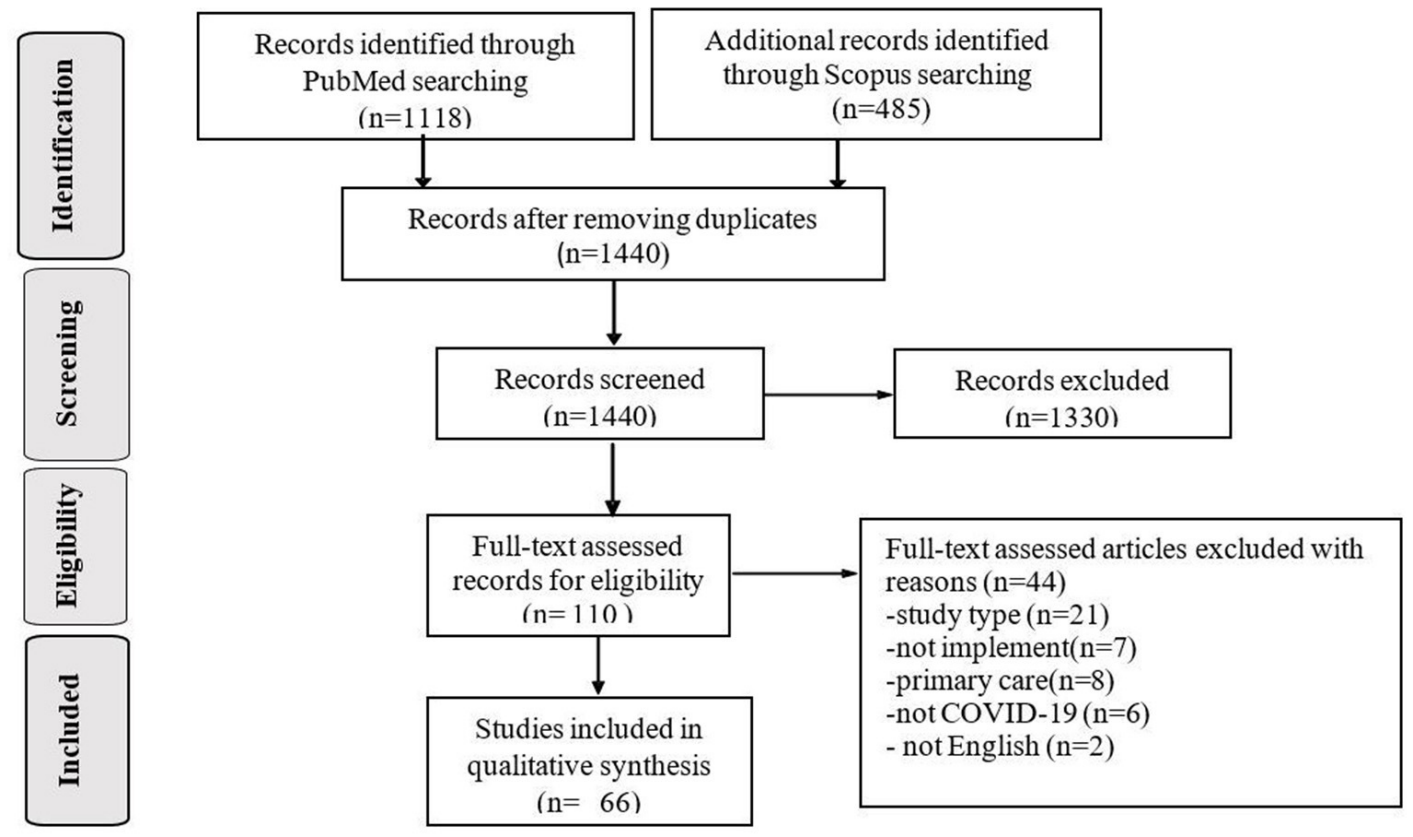
PRISMA Flowchart.

Figure 1 illustrates the articles obtained by searching the literature in a flowchart. A total of 1,602 articles were retrieved, of which 163 were duplicates. After screening, 110 documents were qualified for full-text analysis, and 66 were included in the final analysis.

### Characteristics of telemedicine studies included in this scoping review

**Figure 2:**
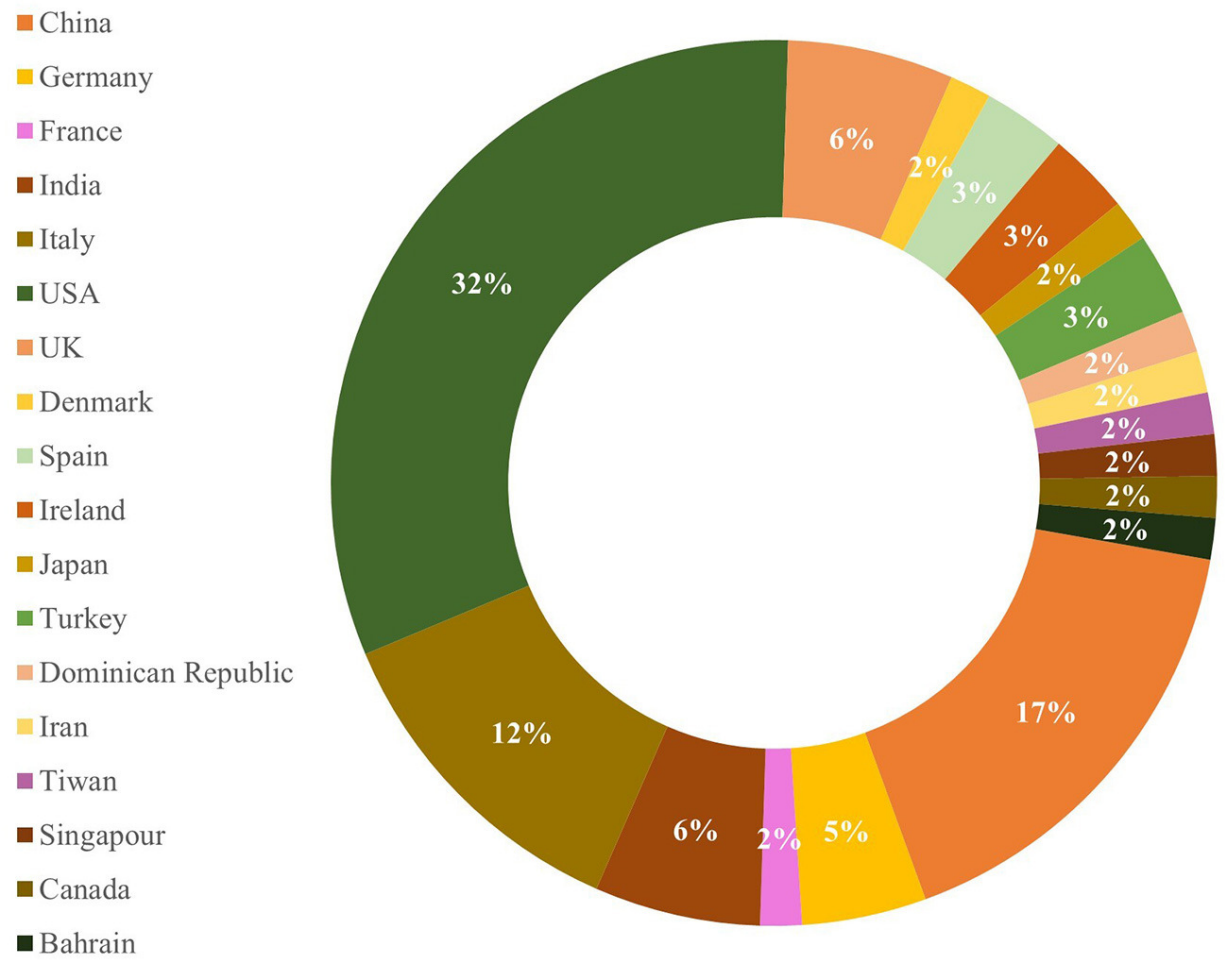
Distribution of the reviewed studies based on country.

All 66 studies reviewed in this research were published in 2020. Out of the 66 articles, 21 (32%) articles were related to the implementation of telemedicine systems in the United States, and the remaining articles were related to telemedicine systems implemented in China (17%), Italy (12%), India (6%), and the United Kingdom (6%) (Figure 2). Regarding the research methods used in these studies, it was found that 49 (74%) articles used a cross-sectional design, 11 (17%) articles used a cohort design, and six (9%) studies used a pre-post comparison design.

### Results related to data charting

The charting results of data related to the four research domains (domains A to D) evaluated in the reviewed studies and the definitions related to each category are presented in Table 2. Most telemedicine studies had functionality (domain A) in the form of counseling (*n* = 59, 90% of the studies). As shown in Table 3, technologies used for counseling included phones, social media platforms, special platforms, videoconferencing, smartphones, and video calls. In addition, seven studies designed telemedicine to diagnose diseases using technologies such as social media platforms, specific platforms, videoconferencing, and phones. In these studies, primary physicians were fully responsible for patients (*n* = 7, 11%). Moreover, in 12 studies, tele-monitoring of patients was performed by phone, designed platforms, social media, Bluetooth devices, and video calls. As shown in Table 3, mentoring was mostly performed using phones, followed by special platforms, social media platforms, Bluetooth, and video calls.

**Table 2:** Results based on data charting.

**Domain and Category**		**Definitions**	**Studies (** ***N*** = **66)**, ***N*****(%)**
Functionality (A)		Contains all aspects of the medical care process.	
Counseling		A remote appointment with a doctor or patient in order to seek advice	59 (90)
Diagnosis		Remote diagnosis by a radiologist, pathologist, cardiologist, or other specialists based on transferred images, records, and laboratory results	7 (11)
Monitoring		Controlling patients at home with various forms of telemetry	12 (18.5)
Mentoring		Giving remote guidance typically by surgeons and other specialists to other surgeons	4 (6)
Technology (B)			
Synchronicity	Synchronously	Concurrent interaction of participants present in different places	50 (76)
	Asynchronously	In the asynchronous (store-and-forward) method, participants do not interact in real-time.	6 (9)
	Mix method	Use of both methods mentioned above	10 (15)
Network design	Social networks	Use of Internet-based social media sites to communicate with patients	19 (29)
	Other networks	Only use internet, a virtual private network, and other tools to communicate with patients	47 (71)
Setting (C)			
Emergency	Providing emergency inptient and outpatient hospital services to patients to prevent death or severe health impairment		4 (7)
In-patient	Providing any services or treatments to patients hospitalized, either for a short time or for a long time.		3 (5)
Outpatient	Providing any services or treatments to non-hospitalized patients		51 (85)
Other	Any care other than those mentioned above		2 (3)
Outcomes (D)			
Healthcare resource utilization	Outcomes that measure the use of healthcare resources		24 (41)
Patient	Outcomes that evaluate prospective and clinical characteristics of patients and their family		27 (47)
Healthcare provider	Outcomes that assesse the function and satisfaction of healthcare providers		7 (12)

**Table 3:** Applied technology in telemedicine systems based on functionality.

	**Phone**	**Video**	**Special**	**Social Media**	**Smart**	**Online**	**Bluetooth**	**Video**	**Multiple**
		**conferencing**	**platform**	**platform**	**phone**			**call**	**technology**
Consultation	16 (27 %)	5 (9 %)	10 (17 %)	14 (24 %)	2 (3 %)	3 (5 %)	-	4 (7 %)	5 (8 %)
Diagnosis	1 (14 %)	1 (14 %)	1 (14 %)	2 (29 %)	1 (14 %)	-	-	-	1 (14 %)
Monitoring	6 (46 %)	-	3 (23 %)	1 (8 %)	-	-	1 (8 %)	1 (8 %)	-
Mentoring	-	-	2 (50 %)	2 (50 %)	-	-	-	-	-

Regarding domain B, telemedicine systems were predominantly implemented synchronously (*n* = 50, 76%), and 10 studies (15%) used both synchronous and asynchronous technologies. Technologies employed in this domain included phones (14 studies), social media platforms (11 studies), special platforms (six studies), videoconferencing (five studies), video calls (seven studies), smartphone (two studies), Bluetooth (one study), and multiple technologies (four studies).

Most telemedicine studies used the Internet and other telemedicine networks. However, a large number of studies used social media networks, such as WhatsApp, Line, and other video and audio communication networks (19 studies).

Out of the 66 studies, 11 (17%) studies reported descriptive results related to domain D and thus were not included in the outcome analysis. A total of 58 outcome variables were evaluated in 55 studies and grouped into three categories (i.e., resource utilization, patient, and healthcare provider outcomes). First, out of the 55 studies, 21 (38%) studies provided data on resource utilization outcomes. Out of the 55 studies, patient and healthcare provider outcomes were reported by 24 (43%) and 6 (11%) studies, respectively. Four remained studies investigated patient and health resource utilization outcomes. Second, 25 studies investigated healthcare resource utilization outcomes, including hospital discharge, visit rate, hospital admission, no-show rate, rate of canceled visit, and stroke alert volumes. The most frequent outcomes evaluated in this category were visit rate and no-show rate, which were investigated in 13 and five studies, respectively. Third, 28 studies evaluated patient outcomes in this review. Patient outcomes evaluated in these studies included usage rate, satisfaction, attitude, acceptance rate, patient compliance, mortality rate, glucose management indicator (GMI), tinnitus handicap inventory scoring, travel time, and total ischemia time (TIT). Results about usage rate and satisfaction were reported in more studies. Forth, in terms of provider outcomes, six studies provided results regarding activity rate, physician and hospital staff satisfaction, managing patients, and accuracy of diagnosis. Physicians' satisfaction was evaluated in four studies.

Regarding domain C, six studies provided no information about the services or care delivered to patients and thus were not included in the setting analysis. Out of the 60 studies, 51 (85%) studies provided outpatient or follow-up services. Three (5%) studies designed telemedicine for inpatient care delivery. Emergency care was delivered in four (7%) studies through telemedicine, and two (3%) studies designed telemedicine for a quarantined traveler and long-term facilities.

### Telemedicine based on care delivery

#### Telemedicine for emergency care

Four studies reported how the telemedicine system was used to address specific events related to emergency care delivery during the COVID-19 pandemic. A recent study used tele-ophthalmology to evaluate eye emergency conditions; in this study, phone, simple smartphone, or web applications were used to deliver care (18). According to the results, the misdiagnosis rate was only 1%, which led to delays in care delivery. Another study implemented a tele-stroke network to assess patients *via* video calls, which led to a decrease in inpatient mortality and stroke alert volumes (19). In addition, pediatric patients with COVID-19 were assessed in a study *via* telephone and then hospitalized if needed (20). In another study, tele-emergency care delivery through a designed platform increased the usage rate of telemedicine and satisfaction in pregnant women (21).

#### Telemedicine for hospitalized patients

Two studies used telemedicine to provide inpatient and outpatient care. These studies were conducted with the aim of tele-monitoring and tele-psychiatry *via* special platforms designed by investigators (22) and the InTouch platform (23), respectively. Another study used a real-time telemetry system in an isolation ward (24).

#### Telemedicine for outpatient care

Telemedicine was implemented in 51 (85%) studies to provide outpatient or follow-up services. Out of the 51 studies, 18 (35%) studies implemented telemedicine systems *via* telephone for counseling and follow-up (25–42). Among which two studies added asynchronous technology (voicemail and website) to the care delivery process (26, 32). 14 outcomes were investigated in these 18 studies (five healthcare resource utilization outcomes, one provider outcome, and eight patient/caregiver outcomes). This technology was effective in improving all provider and patient outcomes and some healthcare resource utilization outcomes. Three studies reported outcomes *via* the number that were not considered (35, 38, 40).

Some patients used messaging applications of social media as an alternative. These mobile applications could help make quick decisions by providing instant communication based on text messages and images. Out of the 51 studies, 13 (25%) studies accepted social media and messaging applications as technologies that could be used in telemedicine systems (43–55). The most widely used online application in five studies was WhatsApp (44, 45, 53–55). WeChat was used in China during the COVID-19 outbreak as a telemedicine communication tool for counseling and disease diagnosis (47, 51, 52). The other applications were Zoom, Skype, and FaceTime (43, 46, 48–50).

Videoconferencing was used in six (12%) studies to facilitate patient–provider communication (56–61). Out of the seven outcomes investigated in these six studies, five outcomes were related to patients, and two outcomes were related to healthcare resource utilization. This technology had no effect on mortality (56) and no-show (58) rates. Five studies used video visits without providing any information about this technology (62–66), which was effective in improving patient and healthcare resource utilization outcomes. Six studies used a special platform to provide telemedicine services (67–72). This technology could improve provider, patient, and healthcare resource utilization outcomes; however, in one case, the use of this platform had no effect on the visit rate (71). Two studies applied smartphones to provide outpatient services *via* online visits (73) and social media (74). Other studies employed online technology. Table 4 shows more details about the outcomes.

**Table 4:** Classification of outcome measures based on clinical care.

**Type of clinical care**	**Outcome categories**	**Outcome**	***N*** **(%)**	**Effect** ***N*** **(%)**	**No effect** ***N*** **(%)**
Outpatient care	Health resource utilization	Hospital discharge	1 (2 %)	1 (2 %)	
		Visit rate	12 (24 %)	10 (20 %)	2 (4)
		Hospital admission	2 (4 %)	2 (4 %)	
		No-show rate	4 (8 %)	3 (6 %)	1 (2 %)
		Rate of canceled visit	1 (2 %)	1 (2 %)	
	Patient	Usage rate	4 (8 %)	2 (4 %)	2 (4 %)
		Satisfaction	11 (22 %)	11 (22 %)	
		Attitude	1 (2 %)	1 (2 %)	
		Acceptance rate	1 (2 %)	1 (2 %)	
		Patient compliance	2 (4 %)	2 (4 %)	
		Mortality rate	1 (2 %)	0 (0 %)	1 (2 %)
		Glucose management indicator (GMI)	1 (2 %)	1 (2 %)	
		Travel time	1 (2 %)	1 (2 %)	
		Tinnitus handicap inventory scoring	1 (2 %)	1 (2 %)	
		Total ischemia time	1 (2 %)	1 (2 %)	
	Provider	Physician and hospital staff satisfaction	4 (8 %)	4(2 %)	
		Managing patient	1 (2 %)	1 (2 %)	
	Sum		49 (100 %)	43 (88 %)	6(12%)
In-patient care	Health resource utilization	Visit rate	1 (33 %)	1 (33 %)	
		No-show rate	1 (33 %)	1 (33 %)	
	Provider	Activity rate	1 (33 %)	1 (33 %)	
	Sum		3 (100 %)	3 (100 %)	
Emergency care	Health resource utilization	Stroke alert volumes	1 (17 %)	1 (17 %)	
		Hospital admission	1 (17 %)	1 (17 %)	
	Patient	Usage rate	1 (17 %)	1 (17 %)	
		Satisfaction	1 (17 %)	1 (17 %)	
		Mortality	1 (17 %)		1 (17 %)
	Provider	Accuracy of diagnose	1 (17 %)	1 (17 %)	
	Sum		6 (100 %)	5 (83 %)	1 (17 %)

#### Other care

Two studies reported the use of social media and video counseling to measure the cost of quarantine (75) and the number of visits to long-term facilities (76).

## Discussion

The COVID-19 pandemic had healthcare systems to suspend or drastically reduce in-person service delivery for non–urgent patients to minimize the various transmissions through this way, which increased the use of alternatives, the best one being telemedicine for maintaining social distancing and limiting contagion. The primary purpose of this scoping review was to present an overview of the literature on telemedicine services in clinical care services during the COVID-19 pandemic.

A total of 66 studies of telemedicine by different modalities emerged in this review. There are still serious gaps in the evidence base for telemedicine. The heterogeneity of studies concerning study designs, populations, locations, and / or measures makes challenges. The type of articles included in our review also varied. A vast majority (49 / 66; 74%) were observational or descriptive articles, with the remainder being cohort studies (18, 19, 33, 48, 51, 56, 57, 59, 60, 63, 75, 77) or before–after studies (40, 58, 78–80).

### Functionalities

Most of the included studies were conducted to reduce the number of patients referring to health centers to receive face-to-face healthcare services like visits, assessments, and care. The most common function in the reviewed studies was counseling. Healthcare workers could contact patients through telecommunication tools like videoconferencing or a simple call to collect their required information and provide further counseling and follow-up services if patients could monitor symptoms at home. The second most common function in the reviewed studies was monitoring (22–24, 28, 31, 32, 37, 39, 65, 74, 78, 80). Regular monitoring of data, such as blood glucose level, respiration rate, and oxygen level, could also be performed through telecommunication tools.

### Technology

Telemedicine could be synchronous or asynchronous. Synchronous telemedicine provides platforms for patients and physicians to exchange vital data simultaneously through a real-time video session. Physicians could also use these platforms to perform remote visual examinations of patients without direct contact. Telemedicine employs a wide range of electronic communication media, ranging from phone and teleconferencing to image-sharing and remote patient surveillance. Diverse technologies could be used for different functions. In this regard, social media platforms and phones could be employed for counseling (77), monitoring, and diagnosis. Telecommunications have been proven to be similar to face-to-face contact when used to promote health and assist in the long-term management of chronic diseases (78). Furthermore, the strategic use of synchronous telemedicine when visual assessment is required may be more effective in improving healthcare resource utilization outcomes (85).

### Setting

Modern technologies, smartphones, and popular mobile applications that provide end-to-end encryption, like WhatsApp and Viber, could be effectively used in telemedicine and could also satisfy patients further through video calls.

The role of telemedicine in managing epidemics and pandemics has been described previously (79), and health systems have expanded this technology in response to COVID-19 to provide outpatient, emergency, and inpatient care. In this review, most of the reviewed studies used this method to provide outpatient care. In this method, real-time interactive visual, textual, audio, and data communication tools are employed to deliver medical care, provide counseling, diagnose diseases, give guidance, transfer medical data, and treat patients. Telemedicine is available in the form of telephone, videoconferencing, and social media platforms. Telemedicine limits exposure to vulnerable patients while simultaneously allowing medical practitioners to provide care. In addition, it allows outpatients to communicate remotely with their physicians and allows physicians to screen patients before they have to refer them to the hospital. This could significantly reduce unnecessary patient visits and encourage patients to quarantine themselves and maintain social distance. Increased use of telemedicine has been shown to reduce in-person visits by two-thirds during the COVID-19 pandemic, which is now declining. These virtual consultations could reduce unnecessary in-person referrals to specialists, waiting times for their feedback, and unnecessary travel.

Store-and-forward is a common technology used in hospital-based telemedicine, especially in radiology departments to send images from smaller hospitals to distant locations for interpretation during nights and weekends. However, the store-and-forward technology was reported in none of the tele-emergency care studies. However, due to the nature of emergency medicine, if images were transmitted to this ward, it was for immediate review and consultation. All studies employed simultaneous audio and video transmission tools. Telemedicine applications in emergency rooms (tele-emergency) are a prime example. In this study, it was found that the most frequently used services were emergency care provided for pregnant women, children, and patients in stroke programs.

### Outcome

The popularity of telemedicine is often due to its ability to improve access to health services while remaining efficient in terms of the resources required. Healthcare resource utilization outcomes, especially visit rate and no-show rate, were variables measured by several studies. Telemedicine was able to improve visit rates for new patients (23, 42, 43, 46, 50, 52, 62, 63, 71, 73) and follow-up rates for previous patients (44, 58, 63, 67). It could increase the rate of visits. Increased healthcare utilization could represent over-care or reflect widespread access to care. These results are consistent with the results of previous studies (79). The most common reason for the decline in telemedicine visits in some studies was the lack of physical examination (45). This review showed a mixed effect of telemedicine on no-show rates. Telemedicine potentially increased the efficiency of healthcare resources by significantly reducing patient no-show rates (23, 41, 62, 67). Due to the nature of COVID-19, the no-show rate increased for surgical providers (58). Telemedicine offers significant benefits to the healthcare system, which strongly supports its widespread utilization during and following the COVID-19 pandemic (80).

Out of 27 patient outcomes, five outcomes were clinical outcomes, such as mortality (2 studies), GMI, TIT, and tinnitus handicap inventory score. Telemedicine was effective in improving GMI, TIT, and tinnitus handicap inventory score using special platform, smartphone, and telephone, respectively, but not in improving mortality. Most studies reported overall satisfaction with telemedicine (20, 26, 27, 29, 32, 40, 43, 48, 50, 65, 76) or compared levels of satisfaction with telemedicine and in-person treatments, such as COVID-19 teleconsultation care (56). This finding is consistent with the findings of other investigations on overall satisfaction with telemedicine in areas such as psychiatry, dermatology, and multi-specialty services (86, 87).

Several studies assessed patient-related clinical outcomes associated with telemedicine (18, 28, 55, 67, 79). However, not all literature supports the positive impacts of telemedicine on patient-related clinical outcomes (18, 55). A review study evaluated the use of real-time “store-and-forward” modalities in various fields of medical services and reported equivocal evidence related to clinical management and telemedicine outcomes (87). Similarly, another review study reported inadequate evidence on the clinical effectiveness of telehealth (81). Telemedicine was shown in another study to potentially increase accessibility to health services *via* removing travel time and cost (55). Telemedicine visits increased patient adherence to treatment by increasing their commitment to telemedicine appointments.

Provider outcomes, such as physician satisfaction, diagnosis accuracy, and patient management, could also be improved by telemedicine. This finding is consistent with the findings of many previous studies in this field (82, 83). Physicians have accepted telemedicine due to time-saving and increased flexibility in scheduling telemedicine visits that modify healthcare delivery.

### Limitation

Similar to any other research, this review also has some limitations. Although a systematic literature review in this study led to the identification of 66 quantitative studies, there are still concerns about methodological quality. These concerns are particularly related to the use of different outcome measures, limited reporting, and retrospective data collection methods (due to the observational nature of many of the included studies). This review included only studies published in English, which might have led to publication (language) bias in study selection due to the omission of other relevant articles published in languages other than English. Future work should further explore barriers and facilitators of telemedicine, implications related to costs and reimbursements, and their impact on care delivery. Some important areas for future research include: clearly delineating the requirements of a telemedicine system for a pandemic and providing evidence of improved patient outcomes.

## Conclusion

This study suggests that telemedicine could be adopted in health emergencies as a convenient, safe, scalable, effective, and green method to provide clinical care. The use of telemedicine in pandemics improves the medical care delivery system, especially for outpatient and emergency care. It potentially could help improve patient, provider, and healthcare outcomes. However, future research is needed to address the requirements of a telemedicine system for a pandemic, the characteristics of successful telemedicine systems, and the outcome measures that should be used to evaluate the clinical care services delivered.

## Author contributions

RG designed this scoping review, search strategy, searched databases, and conducted data analysis and interpretation. RG, MJ, AK, and AD conducted an article screening process. RG and AA drafted the manuscript. All authors reviewed and approved it, contributed to the article and approved the submitted version.

## Conflict of interest

The authors declare that the research was conducted in the absence of any commercial or financial relationships that could be construed as a potential conflict of interest.

## Publisher's note

All claims expressed in this article are solely those of the authors and do not necessarily represent those of their affiliated organizations, or those of the publisher, the editors and the reviewers. Any product that may be evaluated in this article, or claim that may be made by its manufacturer, is not guaranteed or endorsed by the publisher.
