# Supplementary material for: Telemedicine solutions for clinical care delivery during COVID-19 pandemic: A scoping review
Source: Front Public Health. 2022 Jul 22;10:937207. doi: 10.3389/fpubh.2022.937207 (PMC9354887; doi:10.3389/fpubh.2022.937207)
Supplement: Supplementary file 1 [file Table_1.pdf]

Supplementary Material 1

Search Strategy

PubMed	Search: (telemetry[Title/Abstract] OR Telemedicine[Mesh] OR Telemedicine [title/abstract] OR Tele-medicine [title/abstract] OR Mobile health [Title/Abstract] OR "mhealth"[Title/Abstract] OR m-Health[Title/Abstract] OR Telehealth[Title/Abstract] OR Tele-health [title/abstract] OR Telecare [title/abstract] OR eHealth[Title/Abstract] OR e-Health[Title/Abstract]) AND ("severe acute respiratory syndrome coronavirus 2"[Supplementary Concept] OR "Wuhan coronavirus" OR "Wuhan seafood market pneumonia virus" OR "COVID19 virus" OR "COVID-19 virus" OR "coronavirus disease 2019 virus" OR "SARS-CoV-2" OR "SARS2" OR "2019-nCoV" OR "2019 novel coronavirus") AND ((fft[Filter]) AND (humans[Filter]) AND (fft[Filter]) AND (2019/1/1:2020/9/19[pdat]) AND (english[Filter])) AND ((fft[Filter]) AND (2019/1/1:2020/9/19[pdat]) AND (english[Filter])) Filters: Full text, Journal Article, English, from 2019/1/1 - 2020/9/19	1118
Scopus	(TITLE-ABS-KEY ((telemetry OR telemedicine OR tele-medicine OR mobile AND health OR mhealth OR m-health OR telehealth OR tele-health OR telecare OR ehealth OR e-health)) AND TITLE-ABS-KEY ("severe acute respiratory syndrome coronavirus 2" OR "Wuhan coronavirus" OR "Wuhan seafood market pneumonia virus" OR "COVID19 virus" OR "COVID-19 virus" OR "coronavirus disease 2019 virus" OR sars-cov-2 OR sars2 OR 2019-ncov OR "2019 novel coronavirus")) AND (LIMIT-TO (DOCTYPE , "ar")) AND (LIMIT-TO (PUBYEAR , 2020) OR LIMIT-TO (PUBYEAR , 2019)) AND (LIMIT-TO (LANGUAGE , "English"))	486
