# Supplementary material for: Telemedicine solutions for clinical care delivery during COVID-19 pandemic: A scoping review
Source: Front Public Health. 2022 Jul 22;10:937207. doi: 10.3389/fpubh.2022.937207 (PMC9354887; doi:10.3389/fpubh.2022.937207)
Supplement: Supplementary file 2 [file Table_2.pdf]

Supplementary Material 2

General characteristics of included study

NO	**Title**	**Country**	**Study type**	**Aim of study**	**Technology**	**Setting**	**Outcomes**
1	Internet Hospitals Help Prevent and Control the Epidemic of COVID-19 in China: Multicenter User Profiling Study.	China	Cross Sectional	to explore the role of internet hospitals during the prevention and control of the COVID-19 outbreak	Free online / Synchronous	Outpatient	1) visit rate
2	Health services provision of 48 public tertiary dental hospitals during the COVID-19 epidemic in China.	China	Cross Sectional	To assess the status of health services provision of public tertiary dental hospitals during the COVID-19 epidemic	online consultation / Synchronous	Outpatient	1) notification rate<br>2) usage of web-based notice<br>3) penetration rate of online professional consultation
3	Managing Urology Consultations During COVID-19 Pandemic: Application of a Structured Care Pathway	USA	Cross Sectional	To describe and evaluate a risk-stratified triage pathway for inpatient urology consultations	Telephone / Synchronous	Outpatient	1) visit rate
4	Telemedicine Online Visits in Urology During the COVID-19 Pandemic—Potential, Risk Factors, and Patients' Perspective	Germany	Cross Sectional	To explored role of telemedicine in COVID-19 pandemic	Video-conference / Synchronous	Outpatient	1) patient attitude
5	Teleconsultation in primary ophthalmic emergencies during the COVID-19 lockdown in Paris: Experience with 500 patients	France	cohort	To describe ability of teleconsultation to properly indicate a physical consultation for fair diagnosis and treatment in eye emergencies	Call / Synchronous	Emergency*	1) accurate diagnose
6	Tele-consultations and electronic medical records driven remote patient care: Responding to the COVID-19 lockdown in India	India	Cross sectional	To describe the experience of tele-consultations addressed at the center of a multitier ophthalmology hospital network	Online phone or video call / Synchronous	Outpatient	1) usage rate
7	Teleurology in the Time of Covid-19 Pandemic: Here to Stay?	Italy	Cross sectional	To assess the implementation and outcomes of telemedicine	Telephone / Synchronous	Outpatient	1) rate of visits canceled
8	Rapid Utilization of Telehealth in a Comprehensive Cancer Center as a Response to COVID-19: Cross-Sectional Analysis	USA	Cross sectional	To analyze the change in video visit volume and compare patient demographics and appointment data	Video conference/ Synchronous	Outpatient	1) visit rate
9	Application and Preliminary Outcomes of Remote Diagnosis and Treatment During the COVID-19 Outbreak: Retrospective Cohort Study	China	Cohort	To analyze the outcomes and advantages of telemedicine in the context of the COVID-19 outbreak	Online consultation / Synchronous	Outpatient	1) satisfaction
10	Effects of Internet Hospital Consultations on Psychological Burdens and Disease Knowledge During the Early Outbreak of COVID-19 in China: Cross-Sectional Survey Study	China	Cross sectional	To explore the role of internet hospitals in relieving psychological burden and increasing disease knowledge during the early outbreak of the COVID-19 pandemic	Online consultation / Synchronous	In-Patient	1) psychological stress
11	What patients" see "doctors in online fever clinics during COVID-19 in Wuhan?	China	Cross sectional	To describe the patient characteristics of an online fever clinic and explore the most important concerns and question of online patients	Online clinic / Synchronous	Outpatient	1)The most important concerns and question of patients

12	Inpatient Transition to Virtual Care During COVID-19 Pandemic	USA	Before-after	To investigate virtual care for inpatient diabetes management during the COVID-19 crisis and compare it with data before the pandemic	Phone / Synchronous	Outpatient	1) Reduced patient and provider exposure 2) Effective diabetes care
13	Can Tele dentistry Improve the Monitoring of Patients during the Covid-19 Dissemination? A Descriptive Pilot Study	Italy	Cross sectional	To describe the advantages of telemedicine in dental practice during the Covid-19 pandemic	Tele-consultations by sending photos / Asynchronous	Outpatient	1) patient compliance
14	Pivoting to teleconsultation for pediatric ophthalmology and strabismus: Our experience during COVID-19 times	India	Cross sectional	To analyze and report the data of teleconsultations provided to pediatric ophthalmology and strabismus patients during COVID-19	Video call consultation / Synchronous	Outpatient	1) usage rate
15	Application value of vital signs telemetry system for 2019 novel coronavirus disease suspected cases in isolation wards	China	Cross-Sectional	To evaluate a vital signs telemetry system in the isolation wards to improve medical resource consumption and heavier hospital control tasks	Telemetry system in real-time via Bluetooth / Synchronous	Other	1) activity rate
16	Rapid implementation of an evidence-based remote triaging system for assessment of suspected referrals and patients with head and neck cancer on follow-up after treatment during the COVID-19 pandemic: Model for international collaboration.	UK	Cross-Sectional	To demonstrate a remote triaging system for assessment of patients with cancer	Telephone triage / Synchronous	Outpatient	1) discharged directly
17	Telehealth in Uro-oncology Beyond the Pandemic: Toll or Lifesaver?	Germ any	Cohort	To determine patients' perspective on adoption of telehealth as a response to the pandemic	Video conference / Synchronous	Outpatient	1) Acceptance rate
18	Telemedicine and COVID-19: an Observational Study of Rapid Scale Up in a US Academic Medical System	USA	Cross-Sectional	To evaluate growing telemedicine program	Video consultation / Synchronous	In-Patient	1) satisfaction
19	Telemedicine During COVID-19 for Outpatient Sports and Musculoskeletal Medicine Physicians	USA	Cross-Sectional	To describe results from a quality improvement initiative during a rapid adoptive phase of telemedicine during the pandemic	Audio visual / Synchronous	Outpatient	1) satisfaction
20	Telemedicine During the COVID-19 Pandemic: Impact on Care for Rare Cancers	UK	Cross-Sectional	To evaluate the impact of telemedicine on patients, clinicians, and care delivery	Telephone / Synchronous	Outpatient	1) satisfaction
21	Telemedicine in Neurosurgery: Lessons Learned and Transformation of Care During the COVID-19 Pandemic	USA	cohort	To determine the extent of adoption of telemedicine across tumor, vascular, spine, and function neurosurgery and utilization for new patient visits.	Video-conference / Synchronous	Outpatient	1) USAGE RATE
22	Telephone consultation as a substitute for face-to-face consultation during the COVID-19 pandemic	Denmark	Cross-Sectional	To assess the frequency of such telephone consultations and families' evaluations of them in a pediatric outpatient clinic during the initial weeks of the COVID-19 pandemic	Telephone / Synchronous	Outpatient	1) satisfaction
23	The response of the mental health network of the Salamanca area to the COVID-19 pandemic: The role of the telemedicine.	Spain	Cross-Sectional	to detect mental illness and respond to them	telephone/ Synchronous	Outpatient	1) The rate of activity
24	Tele neurology as a Solution for Outpatient Care During the COVID-19 Pandemic	USA	Cross sectional	To describe the implementation and utilization of tele neurology across all neurological subspecialities during the COVID-19 pandemic	Express Care Online (ECO) platform either widely available applications (e.g., Facetime and Google Duo) or telephone only	Outpatient	1) visit rate

25	Patients' Experiences of Remote Neurology Consultations during the COVID-19 Pandemic	Ireland	Cross sectional	to compare patients' opinions about platform to face-to-face consultations in general neurology clinics	audio-only telephone consultation / Synchronous	Outpatient	1) patient attitude
26	Feasibility and safety of urgently initiated maternal telemedicine in response to the spread of COVID-19: A 1-month report	Japan	Cross sectional	to investigate the safety of urgently initiated maternal telemedicine in preventing the spread of the coronavirus	visual communication system Kizuna Web / Asynchronous	Outpatient	1) patient compliance
27	Comparison of Clinical Outcomes in Patients with ST Elevation Myocardial Infarction with Percutaneous Coronary Intervention and the Use of a Telemedicine App Before and After the COVID-19 Pandemic	China	before after	to compare outcomes in patients with STEMI who had percutaneous coronary intervention (PCI) and the use of a telemedicine	The Tiantanzhixin app is a free-of-charge application designated for smart phones /Asynchronous	Outpatient	1) total ischemia time (TIT)
28	Telehealth for delivery of hemophilia comprehensive care during the COVID-19 pandemic	Ireland	Cross sectional	To explore patient and healthcare provider (HCP) experience of telehealth in a European Hemophilia Comprehensive Care Centre.	Blue eye app, video communication,<br>email and SMS / Asynchronous And Synchronous	Outpatient	1) provider satisfaction
29	Telemedicine in Otolaryngology in the COVID-19 Era: Initial Lessons Learned	USA	Cross sectional	to define characterization of the patients who accepted and declined telemedicine otology, head, and neck oncology	Epic Telehealth system / Asynchronous	Outpatient	1) visit rate
30	Telephone surveillance during 2019 novel coronavirus disease: Is it a helpful diagnostic tool for detecting acute pulmonary exacerbations in children with chronic lung disease?	Turkey	Cross sectional	to evaluate the efficiency of telephone visits to determine pulmonary exacerbations and hospitalization rates of children with cystic fibrosis and interstitial lung disease	telephone visits / Synchronous	Outpatient	1)Patient status
31	Feasibility and effectiveness of teleco nsultation in children with epilepsy am idst the ongoing COVID - 19 pandemi c in a resource - limited country	India	Cross sectional	to explore the feasibility and efficacy of advanced telecommunication measures to provide an all-inclusive and precise teleconsultation for children with epilepsy	Voice call, text message, picture/video message via WhatsApp<br>/Asynchronous<br>and<br>Synchronous	Outpatient	1)<br>SAISFACTI ON<br>2)visit rate
32	Effectiveness of teleconsultation use in access to mental health services during the coronavirus disease 2019 pandemic in the Dominican Republic	Dominican Republic	Cross sectional	to determine the effectiveness of teleconsultation, use to increase access to mental health	calls, video calls, and electronic messaging services /<br>Asynchronous and Synchronous	Outpatient	1)Patient status
33	Usefulness of a Telemedicine Tool TELEA in the Management of the COVID-19 Pandemic	Spain	Cross sectional	to evaluate usefulness of telemedicine in the management of the coronavirus disease 2019 (COVID-19) pandemic	TELEA platform (a web-based electronic tool with a tablet, a personal computer, or a smart phone)/ Asynchronous	Outpatient	1) the rate of admit
34	Proficiency of virtual follow - up amo ngst tinnitus patients who underwent i ntratympanic steroid therapy amidst C OVID 19 pandemic	India	Cross sectional	to assess the efficacy and feasibility of virtual follow-up in patients who have undergone intratympanic steroid injection for treatment of tinnitus during COVID-19 pandemic	video calling & telephone / Synchronous	Outpatient	1) Tinnitus handicap inventory scoring
35	Tele oncology or telemedicine for oncology patients during the COVID-19 pandemic: the new normal for breast cancer survivors?	Turkey	Cross sectional	to share the gained experience through the implementation of telemedicine methods during the COVID-19 pandemic in oncology center	video calling, WhatsApp or short messaging service, voice call /<br>Asynchronous and Synchronous	Outpatient	1)Patient status

36	A Report of the Tele psychiatric Evaluation of SARS-CoV-2 Patients	Iran	Cross sectional	to assess psychiatric comorbidities in COVID-19 patients by utilizing telepsychiatry	video chat / Asynchronous	Outpatient	1)Patient status
37	Patient Perception of Telehealth Services for Breast and Gynecologic Oncology Care during the COVID-19 Pandemic A Single Center Survey based Study	USA	Cross sectional	To evaluated the use of telehealth services using the validated Service User Technology Acceptability Questionnaire for outpatient of breast and Gynecology cancer	video-based telehealth visits through the electronic medical system / Synchronous	Outpatient	1)satisfaction
38	Use and Cost-Effectiveness of a Telehealth Service at a Centralized COVID-19 Quarantine Center in Taiwan Cohort Study	Taiwan	Cohort Study	To aim of using telehealth to monitor COVID-19 infections in quarantined Taiwanese travelers and to analyze the cost-effectiveness of the quarantine program	popular social media app called LINE / Synchronous	Other	1)cost of the mandatory quarantine
39	Benefits and barriers to pediatric tele-urology during the COVID-19 pandemic	USA	Cohort Study	To present experience of rapidly integrating the use of Remote Video visits (VV) into a previously in-person only practice in pediatric urology department	video visits platform (VV) platforms: (doxy.me and Face time) / Synchronous	Outpatient	1) usage rate 2)travel time
40	SARS-CoV-2-related rapid reorganization of an epilepsy outpatient clinic from personal appointments to telemedicine services A German single-center experience	Germany	Cross sectional	To analyze the acceptance, feasibility, and satisfaction of the SARS-CoV-2-related conversion from face-to-face to telemedicine appointments from the perspectives of both patients and medical professionals in The Epilepsy Center	a telephone contact / Synchronous	Outpatient	1) satisfaction
41	Delivery of urological services (telemedicine and urgent surgery) during COVID-19 lockdown: experience and lessons learnt from a university hospital in United Kingdom	UK	Cross sectional	To look at a 7- week lockdown activity for all scheduled outpatient clinics and urgent procedures in department of Urology	virtual telephone or video clinic slots via ‘Microsoft Teams’ app (Microsoft, USA) / Synchronous	Outpatient	1) visit rate
42	Impact of Telemedicine on Hospitalization and Mortality Rates in Community Based Hemodialysis Centers in Singapore During the COVID-19 Pandemic	Singapore	Cohort Study-before after	To compare medical outcomes in community-based hemodialysis centers cohort before and after telemedicine was introduced.	Remote to access to medical records and Video conferencing / Synchronous ar asynchronous	Outpatient	1)Hospitalization rates 2)mortality rates
43	A multidisciplinary telemedicine model for management of coronavirus disease 2019 (COVID-19) in obstetrical patients	USA	Cross sectional	To describe the clinical and process outcomes associated with the implementation of a novel multidisciplinary telemedicine surveillance model for the outpatient management of patients with suspected coronavirus disease 2019(COVID-19)	Telephone and voicemail / Synchronous and Asynchronous	Outpatient	1) managing patent
44	Patient Satisfaction with Telemedicine During the COVID-19 Pandemic: Retrospective Cohort Study	USA	Retrospective Cohort Study	to determine if patient satisfaction differs between video and in-person visits	Video / Asynchronous	Outpatient	1)satisfaction
45	Management of cardiac implantable electronic device follow- up in COVID-19 pandemic: Lessons learned during Italian lockdown	Italy	Cohort Study	To report the efficacy and patient satisfaction with the new cardiac implantable electronic devices management protocol adopted during the coronavirus disease-2019 (COVID-19)-related Italian lockdown	Telephone /Synchronous	Outpatient	1)satisfaction

46	Health-care organization for the management and surveillance of SARS-CoV-2 infection in children during pandemic in Campania region, Italy	Italy	Cross sectional	To describe the specific pathway used to manage children with or exposed to COVID 19 infection based on the use of telemedicine	Telephone/Synchronous	Emergency	1)Hospital admission
47	Acute Stroke Presentation, Care, and Outcomes in Community Hospitals in Northern California During the COVID-19	USA	cohort study	to compare temporal trends in volume of acute stroke alerts, patient characteristics, tele stroke care, and short-term outcomes pre- and post-Shelter-in-place orders	Video / Asynchronous	Emergency	1)stroke alert volumes 2)inpatient mortality for stroke.
48	Feasibility and Acceptability of Telemedicine to Substitute Outpatient Rehabilitation Services in the COVID-19 Emergency in Italy: An Observational Everyday Clinical-Life Study	Italy	Cross sectional	To investigate the feasibility and acceptability of telemedicine as a substitute for outpatient services in emergency situations such as the sudden surge of the COVID-19 pandemic in Italy	writing and video via free teleconference apps (Skype, a WhatsApp and Google Meet software / Synchronous	Outpatient	1)The number of services
49	A virtual care program for outpatients diagnosed with COVID-19: a feasibility study	Canada	Cross sectional	to develop and test the feasibility of a virtual care program for self-isolating outpatients diagnosed with COVID-19	telephone / Synchronous	Outpatient	1)The number of patients
50	Adherence and acceptability of telehealth appointments for high-risk obstetrical patients during the coronavirus disease 2019 pandemic	USA	Cross sectional	to describe patient and provider attitudes toward telehealth for the delivery of high-risk obstetrical care	audio-audio or video-audio connection / Synchronous	Outpatient	1)satisfaction 2)no-show
51	Assessing the Impact of a Rapidly Scaled Virtual Urgent Care in New York City During the COVID19 Pandemic	USA	retrospective cohort	to describe and assess the impact of a rapidly scaled virtual 10 urgent care during the COVID-19 pandemic	online video telehealth platform. Epic MyChart application on mobile devices and computers / Synchronous	In-Patient	1)visit rate 2)satisfaction
52	Characteristics of online medical care consultation for pregnant women during the COVID-19 outbreak: cross-sectional study	China	cross-sectional	to described the needs of pregnant women and the contents of online obstetric consultation in representative areas with various severity of the epidemic in China	FREE online communication platform / Synchronous	Emergency	1) usage rate 2)satisfaction
53	Digital Health Technologies Respond to the COVID-19 Pandemic In a Tertiary Hospital in China: Development and Usability Study	China	Before/After	To introduce the practical experience of the design and implementation of a web-based COVID-19 service platform at a tertiary hospital	web-based COVID-19 service platform / Asynchronous	In-Patient	1)Number of screenings, symptom monitoring
54	Glycemic control in people with type 1 diabetes using a hybrid closed loop system and followed by telemedicine during the COVID-19 pandemic in Italy	Italy	retrospective study	To evaluating the metrics of glycemic control in people with type 1 diabetes using the hybrid closed loop (HCL) system during the COVID-19 lockdown	CareLinkTM (Medtronic), platform / Asynchronous	Outpatient	1) glucose management indicator (GMI)
55	Management of differentiated thyroid cancer through nuclear medicine facilities during Covid-19 emergency: the telemedicine challenge	Italy	cross-sectional	To investigate whether a telemedicine service (TMS) carried out during the Covid-19 pandemic impacted on management of patients with differentiated thyroid cancer (DTC).	two active phone numbers, a fax number, and an e-mail address / Asynchronous	Outpatient	1)The number of outpatient visits 2) missing rate
56	Patient care modifications and hospital regulations during the COVID-19 crisis created inequality and functional hazard for patients with orthopedic trauma	UK	cross-sectional	to assess the implications of this management on future orthopedic practice and patient satisfaction of our virtual fracture follow-up clinics	Virtual Fracture Clinic (combination of video and telephone clinics)/ Synchronous	Outpatient	1) average satisfaction
57	Patient Characteristics Associated with Telemedicine Access for Primary and Specialty Ambulatory Care During the COVID-19 Pandemic	USA	cohort	To evaluate whether inequities are present in telemedicine use and video visit use for telemedicine visits during the COVID-19 pandemic	telemedicine visits/ Synchronous and Asynchronous	Outpatient	1) visit rate

58	Pilot Study Using Telemedicine Video Consultation for Vascular Patients' Care During the COVID-19 Period	China	cross-sectional	to evaluate the effectiveness and patients' satisfaction of using telemedicine virtual communications to provide remote health care to vascular patients	Video calls using WeChat software/ Synchronous	Outpatient	1) SATISFACTION
59	Primed for a pandemic: Implementation of telehealth outpatient monitoring for women with mild COVID-19	USA	cross-sectional	to describe the experience and lessons learned while designing and implementing a virtual telemonitoring	Telehealth monitoring/ Asynchronous	Other	1)visit rate<br>2) no-show
60	Rapid Implementation of a COVID-19 Remote Patient Monitoring Program	USA	cross-sectional	To evaluate early lessons from a remote patient monitoring engagement and education technology solution for patients with COVID-19 symptoms.	online e-Visit/ Asynchronous	Outpatient	1)visit rate
61	Rapid Scaling Up of Telehealth Treatment for Tobacco-Dependent Cancer Patients During the COVID-19 Outbreak in New York City	USA.	cross-sectional	To examine the effect of rapid scaling of tobacco treatment telehealth on patient engagement	telehealth visits/ Synchronous	Outpatient	1)visit rate<br>2) no-show
62	Rapid Telehealth-Centered Response to COVID-19 Outbreaks in Post-acute and Long-Term Care Facilities	USA	cross-sectional	to inform PA/LTC facilities and neighboring health care partners how to collaboratively utilize telehealth-centered strategies to improve outcomes in facility outbreaks.	Video Telemedicine Consultation / Synchronous	Other	1) number of consultations
63	Sustainable Diabetes Care Services during COVID-19 Pandemic	Bahrain	cross-sectional	to find an innovative method to sustain effective diabetes care services amidst the COVID-19 pandemic	online video call/ Synchronous	Outpatient	1)visit rate<br>2) no-show
64	Teledermatological monitoring for chronic cutaneous autoimmune diseases with smart working during Covid-19 emergency in a tertiary center in Italy.	Italy	Cross-sectional	to start teledermatological service in smart working using phone calls and emails	telephonic visits and if required store and forward tele dermatology (SAF-TD)/ Synchronous and Asynchronous	Outpatient	1)Patient Visit Volume
65	The Impact of Telemedicine Adoption on a Multidisciplinary Bariatric Surgery Practice During the COVID-19 Pandemic	USA	before after	to show how full-time telemedicine adoption has impacted patient visit volume and attendance in a comprehensive metabolic and weight loss center.	audio-video telehealth visits/ Synchronous	Outpatient	1)visit rate<br>2) no-show
66	The Protective Impact of Telemedicine on Persons with Dementia and Their Caregivers During the COVID-19 Pandemic	China	Before/After	to evaluate whether supplementary telehealth via video-conferencing platforms could bring additional benefits to care-recipient with NCD and their spousal caregivers at home	video communication apps, namely, Zoom, WhatsApp, or FaceTime/ Synchronous	In-Patient	self-efficacy mental health perceived burden quality of life
